# Supplementary material for: Differential effects of light and feeding on circadian organization of peripheral clocks in a forebrain Bmal1 mutant
Source: eLife. 2014 Dec 19;3:e04617. doi: 10.7554/eLife.04617 (PMC4298698; doi:10.7554/eLife.04617)
Supplement: Figure 8—source data 1. — DOI: http://dx.doi.org/10.7554/eLife.04617.017 [file elife04617s002.docx]

Figure 8-source data 1(a). Summary of statistical analysis of circular plots presented in Figure 8.

Mean Phase Angle is the average peak phase of bioluminescence rhythms of each tissue from the indicated sample number. The distribution is shown as circular standard deviation (Circular SD). Circular Variance is calculated as *V* = 1 - *r*, where *r* is the length of mean vector (i.e. the strength of the phase clustering), hence is expressed as values between 0 and 1. Watson-Williams F-test was performed to compare the mean phase angle in each tissue from Fx/Fx and BKO mice under food restriction in DD. Variance (distribution of peak phase values) was compared by bootstrapping to estimate P-values for each comparison.

| **Fx/Fx ZT vs CT** | | Pituitary | Liver | Kidney | Heart | Lung | Spleen |
| --- | --- | --- | --- | --- | --- | --- | --- |
| Fx/Fx  in ZT | Mean Phase Angle (˚) | 200.5  (n = 11) | 150.4  (n = 11) | 148.6  (n = 11) | 194.7  (n = 11) | 180.2  (n = 11) | 216.7  (n = 11) |
|  | Circular SD (˚) | 68.19 | 43.58 | 81.64 | 86.04 | 92.81 | 90.85 |
|  | Circular Variance | 0.507 | 0.251 | 0.638 | 0.676 | 0.731 | 0.716 |
| Fx/Fx  in CT | Mean Phase Angle (˚) | 276.7  (n = 11) | 294.2  (n = 11) | 282.1  (n = 11) | 295.6  (n = 11) | 283.6  (n = 11) | 329.1  (n = 11) |
|  | Circular SD (˚) | 42.47 | 49.67 | 13.08 | 16.37 | 16.30 | 12.82 |
|  | Circular Variance | 0.240 | 0.313 | 0.026 | 0.040 | 0.040 | 0.025 |
| Watson-Williams F-test | |  |  |  |  |  |  |
|  | P value | **0.009** | **2.98E-6** | **2.02E-4** | **0.003** | **0.006** | **0.003** |
| Comparison of variance | |  |  |  |  |  |  |
|  | P value for ZT > CT | 0.0739 | 0.6135 | **0.0004** | **0.0001** | **0.0001** | **<0.00001** |
| **Fx/Fx CT vs BKO** | | Pituitary | Liver | Kidney | Heart | Lung | Spleen |
| BKO | Mean Phase Angle (˚) | 227.2  (n = 11) | 150.1  (n = 11) | 71.80  (n = 11) | 46.19  (n = 11) | 8.759  (n = 11) | 73.28  (n = 11) |
|  | Circular SD (˚) | 46.41 | 14.77 | 9.454 | 51.63 | 30.80 | 49.55 |
|  | Circular Variance | 0.280 | 0.033 | 0.014 | 0.334 | 0.135 | 0.312 |
| Watson-Williams F-test | |  |  |  |  |  |  |
|  | P value | **0.023** | **6.04E-8** | **< 1E-12** | **3.40E-6** | **2.29E-7** | **3.13E-6** |
| Comparison of variance | |  |  |  |  |  |  |
|  | P value for Fx/Fx > BKO |  | **0.0008** | 0.1153 |  |  |  |
|  | P value for Fx/Fx < BKO | 0.3960 |  |  | **0.0004** | **0.0333** | **0.0001** |
| **Fx/Fx ZT vs BKO** | | Pituitary | Liver | Kidney | Heart | Lung | Spleen |
| Watson-Williams F-test | |  |  |  |  |  |  |
|  | P value | 0.329 | 0.978 | **0.01** | **9.81E-4** | **7.68E-4** | **0.002** |
| Comparison of variance | |  |  |  |  |  |  |
|  | P value for Fx/Fx > BKO | 0.0682 | **0.0008** | **0.0003** | 0.0624 | **0.0010** | **0.0406** |

Figure 8-source data (b). Statistical analysis of circular variance for peak bioluminescence in individual mice under restriction feeding. Shown are mean vector, circular SD, and circular variance of pituitary, liver, kidney, heart, lung, spleen in an individual mouse. Mann Whitney test for circular variances between Fx/Fx WT control and BKO mice shows P = 0.0002.

| **FR** | Mean Vector (˚) | Circular SD (˚) | Circular Variance | **FR** | Mean Vector (˚) | Circular SD (˚) | Circular Variance |
| --- | --- | --- | --- | --- | --- | --- | --- |
| WT1 | 291.291 | 18.314 | 0.050 | BKO1 | 74.870 | 75.297 | 0.578 |
| WT2 | 314.873 | 20.798 | 0.064 | BKO2 | 119.369 | 86.127 | 0.677 |
| WT3 | 287.688 | 48.681 | 0.303 | BKO3 | 76.425 | 66.332 | 0.488 |
| WT4 | 290.607 | 17.553 | 0.046 | BKO4 | 100.440 | 56.031 | 0.380 |
| WT5 | 295.641 | 29.002 | 0.120 | BKO5 | 3.726 | 98.923 | 0.775 |
| WT6 | 290.636 | 32.150 | 0.146 | BKO6 | 341.806 | 74.937 | 0.575 |
| WT7 | 282.086 | 48.633 | 0.302 | BKO7 | 344.984 | 80.356 | 0.626 |
| WT8 | 292.279 | 15.208 | 0.035 | BKO8 | 84.430 | 56.341 | 0.383 |
| WT9 | 292.249 | 23.201 | 0.079 | BKO9 | 110.901 | 45.547 | 0.271 |
| WT10 | 307.458 | 22.848 | 0.076 | BKO10 | 91.736 | 57.863 | 0.399 |
| WT11 | 278.987 | 53.688 | 0.355 | BKO11 | 70.109 | 65.468 | 0.479 |
