# Supplementary material for: Differential effects of light and feeding on circadian organization of peripheral clocks in a forebrain Bmal1 mutant
Source: eLife. 2014 Dec 19;3:e04617. doi: 10.7554/eLife.04617 (PMC4298698; doi:10.7554/eLife.04617)
Supplement: Figure 9—source data 1. — DOI: http://dx.doi.org/10.7554/eLife.04617.019 [file elife04617s003.docx]

Figure 9-source data (a). Summary of statistical comparison of peak phases from Fx/Fx mice under different conditions.

All parameters were presented in previous Tables. Watson-Williams F-test was performed to compare the mean phase angle in each tissue from Fx/Fx mice under different conditions. Variance (distribution of peak phase values) was compared by bootstrapping to estimate P-values for each comparison.

| **Fx/Fx LD vs DD** | | Pituitary | Liver | Kidney | Heart | Lung | Spleen |
| --- | --- | --- | --- | --- | --- | --- | --- |
| Watson-Williams F-test | |  |  |  |  |  |  |
| P value |  | 0.106 | **5.30E-4** | **0.007** | **0.005** | **0.040** | 0.167 |
| Comparison of variance | |  |  |  |  |  |  |
| P value | for LD > DD | **0.0199** |  | 0.1321 |  | 0.3119 | **0.0434** |
|  | for LD < DD |  | **0.0137** |  | 0.1194 |  |  |
| **Fx/Fx LD vs DD+FR ZT** | | Pituitary | Liver | Kidney | Heart | Lung | Spleen |
| Watson-Williams F-test | |  |  |  |  |  |  |
| P value |  | **0.020** | **3.42E-4** | **9.18E-4** | **0.020** | **0.009** | **0.003** |
| Comparison of variance | |  |  |  |  |  |  |
| P value | for LD < DD+FR | **0.0025** | **0.0020** | **0.0020** | **0.0001** | **0.0001** | **<0.00001** |
| **Fx/Fx DD vs DD+FR CT** | | Pituitary | Liver | Kidney | Heart | Lung | Spleen |
| Watson-Williams F-test | |  |  |  |  |  |  |
| P value |  | 0.356 | **3.51E-4** | **6.09E-4** | **0.005** | 0.444 | 0.152 |
| Comparison of variance | |  |  |  |  |  |  |
| P value | for DD < DD+FR | **0.0215** | **0.0253** | 0.4905 | 0.5278 | 0.4054 | 0.1366 |

Figure 9-source data (b). Summary of statistical comparison of peak phases from BKO mice under different conditions.

All parameters were presented in previous Tables. Watson-Williams F-test was performed to compare the mean phase angle in each tissue from BKO mice under different conditions. Variance (distribution of peak phase values) was compared by bootstrapping to estimate P-values for each comparison.

| **BKO LD vs DD** | | Pituitary | Liver | Kidney | Heart | Lung | Spleen |
| --- | --- | --- | --- | --- | --- | --- | --- |
| Watson-Williams F-test | |  |  |  |  |  |  |
| P value |  | 0.173 | 0.309 | **0.030** | 0.113 | 0.170 | 0.672 |
| Comparison of variance | |  |  |  |  |  |  |
| P value | for LD < DD | **0.0150** | **<0.00001** | **0.0009** | **0.0083** | **0.0190** | **0.0118** |
| **BKO LD vs DD+FR** | | Pituitary | Liver | Kidney | Heart | Lung | Spleen |
| Watson-Williams F-test | |  |  |  |  |  |  |
| P value |  | **0.007** | **1.28E-7** | **< 1E-12** | **5.09E-9** | **4.07E-10** | **2.58E-7** |
| Comparison of variance | |  |  |  |  |  |  |
| P value | for LD > DD+FR |  | 0.4077 | **0.0179** |  |  |  |
|  | for LD < DD+FR | **0.0483** |  |  | **0.0018** | 0.0527 | **0.0020** |
| **BKO DD vs DD+FR** | | Pituitary | Liver | Kidney | Heart | Lung | Spleen |
| Watson-Williams F-test | |  |  |  |  |  |  |
| P value |  | 0.301 | 0.246 | **3.39E-9** | **2.03E-7** | **2.50E-7** | **7.00E-6** |
| Comparison of variance | |  |  |  |  |  |  |
| P value | for DD > DD+FR | 0.1857 | **<0.00001** | <**0.00001** | 0.5628 | 0.4693 | 0.8642 |
